# Effect of a Medicinal *Agaricus blazei* Murill-Based Mushroom Extract, AndoSan^™^, on Symptoms, Fatigue and Quality of Life in Patients with Ulcerative Colitis in a Randomized Single-Blinded Placebo Controlled Study

**DOI:** 10.1371/journal.pone.0150191

**Published:** 2016-03-02

**Authors:** Stig Palm Therkelsen, Geir Hetland, Torstein Lyberg, Idar Lygren, Egil Johnson

**Affiliations:** 1 Department of Gastrointestinal and Pediatric Surgery, Oslo University Hospital, Ullevål, Norway; 2 Immunology and Transfusion Medicine, Oslo University Hospital, Ullevål, Norway; 3 Medical Biochemistry, Oslo University Hospital, Ullevål, Norway; 4 Medicine, Oslo University Hospital, Ullevål, Norway; 5 Faculty of Medicine, University of Oslo, Norway; University Hospital Llandough, UNITED KINGDOM

## Abstract

**Background:**

Ingestion of AndoSan^™^, based on the mushroom *Agaricus blazei* Murill, has previously been shown to exhibit anti-inflammatory effects because of reduction of pro-inflammatory cytokines in healthy individuals and patients with ulcerative colitis. In this randomized single-blinded placebo controlled study we examined whether intake of AndoSan^™^ also resulted in clinical effects.

**Methods and Findings:**

50 patients with symptomatic ulcerative colitis were block-randomized and blinded for oral daily intake of AndoSan^™^ or placebo for the 21 days’ experimental period. The patients reported scores for symptoms, fatigue and health related quality of life (HRQoL) at days 0, 14 and 21. Fecal calprotectin and general blood parameters were also analyzed. In the AndoSan^™^ group (n = 24) symptoms improved from baseline (day 0) to days 14 and 21, with respective mean scores (95% CI) of 5.88 (4.92–6.83), 4.71 (3.90–5.52) (p = 0.002) and 4.50 (3.70–5.30) (p = 0.001). Corresponding improved mean scores (±SD) for total fatigue were 16.6 (5.59), 14.1 (4.50) (p = 0.001) and 15.1 (4.09) (p = 0.023). These scores in the placebo group (n = 26) were not improved. When comparing the two study groups using mixed model statistics, we found significant better scores for the AndoSan^™^-patients. HRQoL for dimensions bodily pain, vitality, social functioning and mental health improved in the AndoSan^™^ group. There were no alterations in general blood samples and fecal calprotectin.

**Conclusions:**

Beneficiary effects on symptoms, fatigue and HRQoL from AndoSan^™^ consumption were demonstrated in this per-protocol study, supporting its use as a supplement to conventional medication for patients with mild to moderate symptoms from ulcerative colitis. The patients did not report any harms or unintended effects of AndoSan^™^ in this study.

**Trial Registration:**

ClinicalTrials.gov NCT01496053

## 1. Introduction

*Agaricus blazei* Murill, a mushroom of the *Basidiomycetes* family, grows in the wild in the Piedade area outside of São Paulo, Brazil, and the local population has for centuries utilized it as a health food ingredient. Serious diseases such as atherosclerosis, hyperlipidemia, diabetes and cancer were less prevalent in the Piedade population compared with counterparts in neighboring regions [1], presumably owing to consumption of AbM. The mushroom was brought to Japan in 1966 and introduced to the health food market and effects of AbM (Himematsutake, *jp*) and other *Basidiomycetes* mushrooms such as *Hericium erinaceus* (He) (Yamabushitake, *jp*) [2] and *Grifola frondosa* (Gf) (Maitake, *jp*) [3] have received an increasing research effort.

AbM *per se* and the AbM based mushroom extract, AndoSan^™^, (ACE Co. Ltd., Gifu-ken, Japan), composed of AbM (82.4%), He (14.7%) and Gf (2.9%), contain immunomodulatory ß-glucans but also other biologically active substances like α-glucans [4], proteoglucans [5], lectins [6], ergosterol (provitamin D2) [7], agaritine [8], isoflavonoids [9], anti-oxidant substances [10], and anti-inflammatory substances such as isolated alkaline and aqueous extracts [11] and the steroid 4-hydroxy-17-methylincisterol (4-HM) [12].

AbM *per se* and the AbM based extract, AndoSan^™^, have been shown to exhibit multiple biological effects including anti-tumor, anti-allergic and both pro-inflammatory and anti-inflammatory effects as reviewed [13, 14]. AbM stimulation *in vitro* of mononuclear phagocytes induced secretion of nitric oxide [15] and pro-inflammatory cytokines IL-1ß, Il-6 and TNFα and IL-8 using AndoSan^™^ [16], which in monocyte-derived dendritic cells also stimulated such cytokine production as well as that of chemokine MIP-1ß [17]. One mechanism behind these effects is probably mediated by binding of glucans in the extract to Toll-like receptor 2 [18] as well as to the dectin-1 receptor [19], the lectin-binding site of CD11b/18 [20] and possibly CR4 CD11c/18 [21]. However, since AndoSan^™^, which is an extract of the mushrooms´ mycelium and not their fruit bodies, recently was shown to contain less ß-glucan than anticipated from the published data of ß-glucan content in the fruit bodies [22], action also of other yet not identified immunomodulating substances in the extract must part-take to render the observed effects. The *in vitro* results above were supported by microarray expression analysis in AbM stimulated promonocytic THP-1 tumor cells [23], demonstrating markedly upregulated genes for IL-1ß, IL-8, moderately for TLR-2 and co-operative molecule MyD88, but not for TLR-4. However, in another *in vivo* study, daily consumption of 60 ml of AndoSan^™^ for a week in chronic hepatitis C patients [24] had no effect on expression of these genes in blood cells.

*Ex vivo* stimulation of whole blood with this AbM-based mushroom extract resulted in a pronounced release, mainly from monocytes, of many cytokines being pro-inflammatory (IL-1ß, IL-6, TNFα), anti-inflammatory (IL-10), chemokines (IL-8, MIP-1ß, MCP-1, leukocyte growth factors (G-CSG, GM-CSF), pleiotropic (IL-7, IL-17) as well as of the Th1- (IFNγ, IL-2, IL-12) and Th-2 types (IL-4, IL-5, IL-13) [25]. However, after daily consumption of 60 ml of AndoSan^™^ for 12 days in 8 healthy volunteers, there was a significant reduction in cytokine levels in plasma of IL-1ß, TNFα, IL-6, IL-2 and IL-17, whilst levels of the remaining 12 cytokines in the kit were unaltered, thereby pointing to an anti-inflammatory effect *in vivo*, when given by the oral route.

In patients with ulcerative colitis (UC), increased mucosal levels have been demonstrated for MIP-1ß, MCP-1 and IL-8 [26], IL-1ß [27], IL-6 and TNFα [28]. Cytokine levels in serum are less well studied but increased levels have been reported for IL-6 [29], TNFα [30, 31] and MIP-1ß [32]. Moreover, in a recent extensive review [33] the cytokines eotaxin, GRO (chemokine), TNFα and IL-8 were considered to be persistently elevated in blood of UC patients compared with findings in healthy individuals.

In 10 patients with UC who likewise consumed the mushroom extract, there was an anti-inflammatory cytokine effect [34] as demonstrated by reduction at day 12 from baseline values of *in vivo* levels of one cytokine (MCP-1-ß) in untreated blood and of 7 other cytokines (MIP-1ß, IL-6, IL-1ß, IL-8, G-CSF, MCP-1, GM-CSF) in LPS-stimulated blood *ex vivo*. The level of fecal calprotectin was also reduced as a consequence of consumption of the mushroom extract. Accordingly, the next step was to examine whether a decline in pathological levels of cytokines mediated by the mushroom extract *in vivo*, does result in a putative beneficial clinical effect in patients with UC.

The aim of the present study was to examine whether consumption of AndoSan^™^ for 21 days had a positive impact in patients with UC on clinical sympoms, fatique and quality of life in a randomized single-blinded placebo controlled study. It should be noted that this trial also includes Crohn's disease patients and that those results are being reported separately.

## 2. Materials and Methods

### 2.1. Reagents

The mushroom extract AndoSan^™^ was provided by the company Immunopharma AS (organization no. 994924273), Oslo, Norway. It was stored at 4°C in metal cans and used under sterile conditions *ex vivo* and kept sterile until taken by volunteers for *in vivo* experiments. This mushroom extract is a commercial product produced by the company ACE Co. Ltd., Gifu-ken, Japan, and its extract contained a business secret, part of which has not been revealed until recently. The AbM mixed powder contains per 100 g the following constituents: moisture 5.8 g, protein 2.6 g, fat 0.3 g, carbohydrates 89.4 g, of which ß-glucan constitutes 2.8 g, and ash 1.9 g. The AndoSan^™^ extract contains 82.4% of *Basidiomycetes* mushroom derived from AbM (Himematsutake, *jp*), 14.7% from Hr (Yamabushitake) [2] and 2.9% from Gf (Maitake) [3], and its final concentration was 340 g / l. The amount per liter of the extract was for sodium 11 mg, phosphorus 254 mg, calcium 35 mg, potassium 483 mg, magnesium 99 mg and zinc 60 mg. The LPS content of AndoSan^™^ was found, using the Limulus amebocyte lysate test (COAMATIC Chromo-LAL; Chromogenix, Falmouth, MA, USA) with detection limit 0.005 EU / ml (1 EU = 0.1 ng / ml), to be a miniscule concentration of <0.5 pg / ml. The results from tests for heavy metals were conformable with strict Japanese regulations for health foods. AndoSan^™^ had been heat-sterilized (124°C for 1 h) by the producer. Potential radioactivity in the extract was not detected by examination of the Norwegian Food Safety Authorities.

### 2.2. Analyses

Blood was harvested from the antecubital vein into glass tubes containing 15 IU heparin per ml or 10 mmol EDTA per ml. The EDTA blood was each time (days 0, 14 and 21) analyzed for haemoglobin, haematocrite, mean cellular volume, mean cellular haemoglobin, reticulocytes, immature reticulocytes, leukocytes including a differential count of neutrophils, basophils, eosinophils, lymphocytes and monocytes, thrombocytes, C-reactive protein (CRP), urea, creatinine, bilirubin, aspartate aminotransferase, alanine aminotransferase, lactate dehydrogenase, γ-glutamine transferase, alkaline phosphatase and pancreatic amylase. Fecal calprotectin concentrations (mg/kg) (normal value <50 mg/kg) at days 0, 14, 21 were determined in duplicates as reported [25, 35].

The mainly patient-reported symptom score was a modified version of the Clinical Activity Index (CAI), including only the 4 clinical items and adding one item defining stool consistency (normal = 0, soft = 1, watery = 2) [36]. The modified CAI contained four self-reported items concerning abdominal pain (score 0–3) and stool with regard to frequency (0–4), consistency (0–2) and blood (0–3). The fifth item evaluated by the physician dealt with general well-being (0–3) of the patient. The symptom score ranged from 0–15. Scores 0–2 meant patients in remission and 3–15 gradually increasing disease activity. The modified CAI, rather than the simple clinical colitis activity index (SCCAI) as described in the study-protocol, was used because of recommendation from the participating gastroenterologist in this study.

Self reported health-related quality of life (HRQoL) was assessed with the short form 36 (IQOLA SF-36 Norwegian Version 1.2), which is a generic HRQoL questionnaire consisting of 36 items, of which 35 are grouped into the following eight health domains: (1) physical functioning (PF), (2) social functioning (SF), (3) role limitations due to physical problems (RP), (4) role limitation due to emotional problems (RE), (5) mental health (MH), (6) vitality (VT), (7) bodily pain (BP) and (8) general health perception (GH). Each domain is graded on a scale of 0–100, and the higher the score the better the HRQoL. The validity and reliability of the SF-36 form have been demonstrated for a number of countries including Norway (version 1) [37]. The data from our study were compared with published norms from 2323 individuals in the general population. Only 11 out of 5400 HRQoL questions were unanswered and, accordingly, 10 out of 1200 dimensions were lacking. Using a scoring algorithm for missing data outlined in the SF 36 survey manual, these 10 dimensions could also be included in the results.

Total fatigue score consists of 11 items of graded questions with score 0–3 per question, which is the sum of physical fatigue (7 items) and mental fatigue (4 items). This score has been validated in a Norwegian general population [38]. The respective scores for total, mental and physical fatigue are 0–33, 0–21 and 0–12, and the higher score the more fatigue. The items of physical (1–7) and mental (8–11) fatigue were: 1) Do you have problems with tiredness? 2) Do you need to rest more? 3) Do you feel sleepy or drowsy? 4) Do you have problems with starting things? 5) Are you lacking in energy? 6) Do you have less strength in your muscles? 7) Do you feel weak? 8) Do you have difficulty concentrating? 9) Do you have problems thinking clearly? 10) Do you make slips of the tongue when speaking? 11) How is your memory?

Rectosigmoidoscopy with biopsies, as described in the study-protocol, were not performed because of lack of resources.

### 2.3. Inclusion and Exclusion of Patients

**Fig 1:**
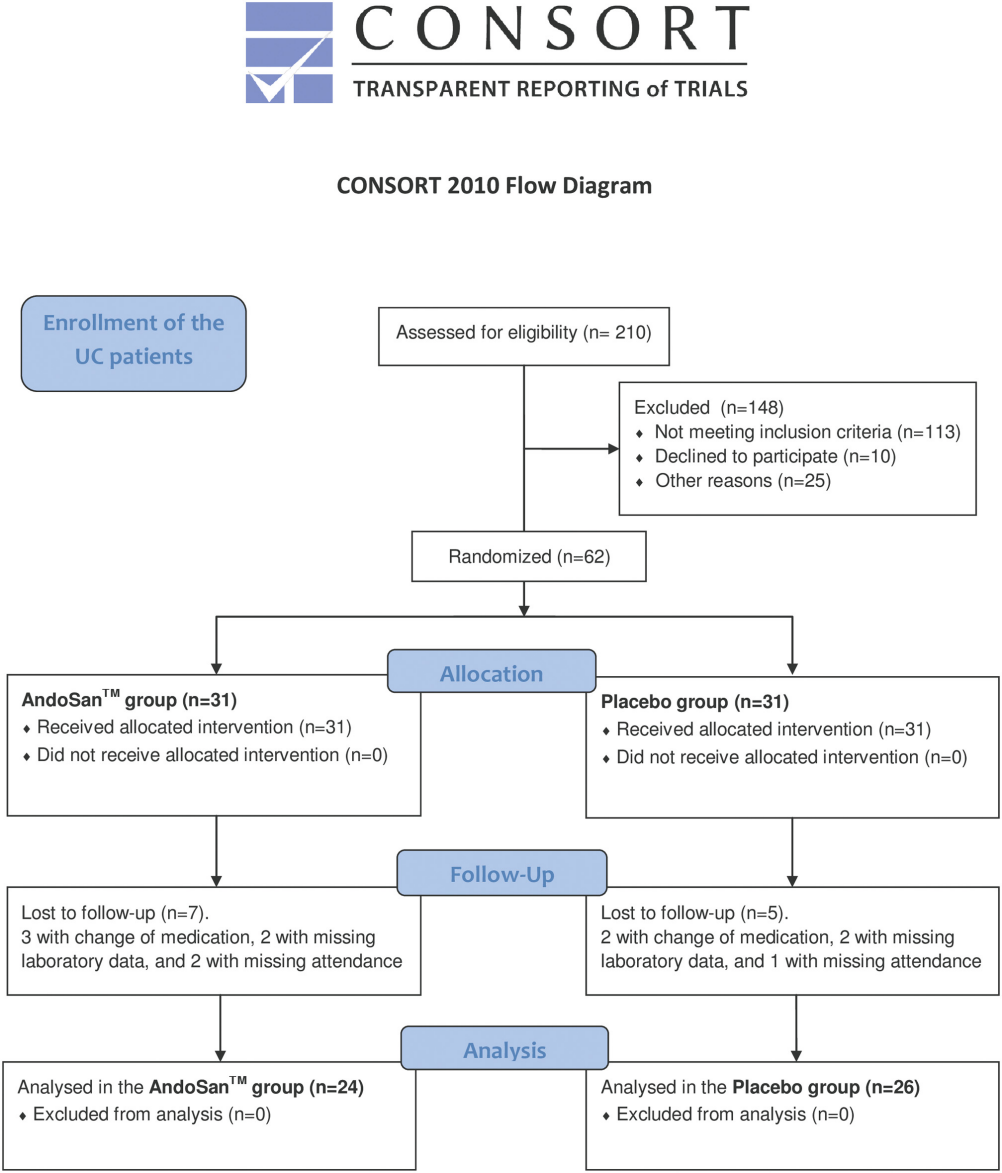
An algorithm showing the scheme for inclusion of the patients in the study.

210 patients with UC were phone interviewed and those with CAI score of at least 3 were given the opportunity to join the study. At the first attendance CAI was re-recorded and criteria for exclusion were pregnancy, biological treatment with antibodies to TNFα (Adalimumab, Infliximab), daily use of more than 5 mg of prednisolone, change of medication and/or consumption of mushroom products from two weeks before till end of the study. A flow chart reveals additional reasons for exclusions (Fig 1).

### 2.4. Experimental Design and Randomization

This is a single-center randomized two-armed patient-blinded study designed to determine whether daily oral intake of a mushroom extract, AndoSan^™^, improved clinical symptoms, fatigue and quality of life in patients with UC during the 21 days’ study period. The patients were evaluated before (visit 1, day 0), during (visit 2, day 14) and after (visit 3, day 21) AndoSan^™^ or placebo consumption (30 ml twice daily). This dose (60 ml/day) reduced levels of pro-inflammatory cytokines and chemokines in healthy volunteres [25] and in patients with UC and CD [34], whilst half dosage (30 ml/day) had no detectable effects (unpublished data). The placebo group was evaluated likewise but received an equal volume of color-like drink with ionized water containing 0.5 ml per liter of caramel color (E150c) with salt.

The 50 patients were divided into 13 groups and manually randomized with the overall allocation ratio of one to one. Block-randomization was done after the phone interview, with uneven and even numbers given for AndoSan^™^ or placebo, respectively. The patients, one by one, were placed in one pile, and the group affiliations were placed in another pile. The randomization was performed by combining one selection from each pile, both anonymized. The first author performed the randomization, enrolled the participants, and assigned participants to interventions. A few patients were excluded throughout the study by not attending or because of intercurrent incidents (disease, unexpected life events). Accordingly, a slight imbalance of the study groups occurred that was corrected in the latter rounds of randomization. More specifically, the 50 UC patients were divided into 13 study groups (range 2–9 per group), each with a study period of 3 weeks. The included 50 symptomatic patients had no missing data and were randomized and blinded for oral daily consumption (30 ml twice daily) of AndoSan^™^ or placebo for the 21 days’ experimental period. Patients in the AndoSan^™^ group and the placebo group self-reported, in written, at visit 1 (day 0), visit 2 (day 14) and visit 3 (day 21) regarding symptoms, fatigue and health-related quality of life. Patient derived blood samples and fecal calprotectin from these visits were also analyzed. All data were stored in a secure database (Access—Microsoft Office) at a server at Oslo University Hospital, Ullevål, Norway. A study number anonymized the patients.

This study is a follow up of a previous pilot study [34] in which there was a reduction of pro-inflammatory cytokines and chemokines in patients receiving the same daily dose of AndoSanTM, but for 12 days. We calculated for prospective differences of 20% between the experimental and placebo group and assumed standard deviation of 20% for the different parameters with a significant level of 5% and a power of 90% (ß = 0.10), demands about 25 patients per randomized arm (calculated in cooperation Oslo Center for Biostatistics and Epidemiology, Oslo University Hospital).

### 2.5. Patient Characteristics

Clinical disease data concerning duration, anatomic extent and activity were registered. The study groups were comparable and with no significant differences with respect to details of demographic data and patient characteristics (Table 1). Eight patients in the AndoSan^™^ group used several medications, Azathioprine and 5-ASA in 4 and Azathioprine and Prednisolone in 1 patient(s), respectively. Three patients used oral 5-ASA combined with rectal enema with Budesonide, Prednisolone and 5-ASA, respectively. In the placebo group 2 patients used Azathioprine and 5-ASA and 5 patients used 5-ASA by oral and rectal route. The medication was unaltered from baseline and throughout the study period, in both groups.

**Table 1:** Demographic and patient data.

	AndoSan^™^	Placebo	P
**Number**	24	26	ns
**Age** (years)	40.5 (23–56)	40.0 (23–67)	ns
**Gender** (male, female)	13, 11	12, 14	ns
**Duration of diagnosis** (months)	96 (4–324)	66 (4–260)	ns
**Anatomic extent**			
rectosigmoidal	5	7	ns
left colon	6	10	ns
entire colon	13	9	ns
**Disease pattern**			
continous	4	2	ns
episodic	20	24	ns
**Medication**			
5-ASA	13	17	ns
Azathioprin	2	0	ns
Several medications	8	7	ns
No medication	1	2	ns
**Comorbidity**			
Arthralgia	8	6	ns
Sarcoidosis	0	1	ns
Diabetes mellitus type 2	1	0	ns
PSC, livercirrhosis and anemia	0	1	ns
Myalgic encephalomyelitis	1	0	ns

Values for age and duration of diagnosis are given as median (range).

### 2.6. Statistical Analysis

Data are presented as mean and standard deviation or as median and range values. Paired sample t-test and Wilcoxon test are used for within-group analysis. The judgement of whether the distributions of the main efficacy variables were so close to the normal distribution that normality-based significance tests may be used, is based on the finding in a paper by Fagerland and Sandvik [39]. Mixed models corrected for baseline values were used for measuring P values between the study groups. P values below 0.05 were considered statistically significant. The SPSS statistical program for the social sciences, version 22 (IBM), was used in the analyses.

### 2.7. Ethical Considerations

The study was approved on April 8, 2011, by the regional ethics committee (REC—South East Norway, ref. 2011/404) and followed the guidelines of the Helsinki declaration. The participants were informed and signed a written consent for participation, including the option of study withdrawal. The patients were recruited and followed up at the department of Medicine, Oslo University Hospital, Ullevål, Norway, in the period of June 2012 to May 2014. The study was registered with unique protocol ID AbM2012-IBD and clinical trials gov ID NCT 01496053 (December 15, 2011). The authors confirm that all ongoing and related trials for this drug/intervention are registered.

## 3. Results

### 3.1. Exclusion of randomized patients

A total of 62 patients, 31 in both study groups, were randomized for inclusion in this study. 12 of these patients were excluded according to the criteria of the study protocol, because three and two changed their medical treatment just prior to or during the study period, two in each group had missing laboratory data, and two and one had missing attendance, in the AndoSan^™^ and placebo group, respectively. Thereby we ended up with 24 patients in the AndoSan^™^ group and 26 in the placebo group.

### 3.2. Age and Gender

Median age for the 50 included patients with UC was 40.5 years (range 23–67). There were 13 men and 11 women in the AndoSan^™^ group and 12 men and 14 women in the placebo group. Respective ages in the two groups were median 35 (range 23–64) and 41.5 (27–50) for men (p = 0.611) and 44 (24–67) and 35.5 (23–56) for women (p = 0.075).

### 3.3. Symptom Score

The symptom scores using the CAI were similar at inclusion in the AndoSan^™^ and placebo groups, with respective mean scores of 5.88 and 5.81. Compared with baseline only the patients in the AndoSan^™^ group reported a significant reduction of symptoms that was also further reduced from visit 2 (day 14) to visit 3 (day 21) after the mushroom extract intake (Table 2). There were no significant differences in baseline symptom scores between male and females within the two groups. When comparing the two groups using mixed models corrected for baseline values, there also was a significant difference (p = 0.023) in favor of the AndoSan^™^ group.

**Table 2:** Symptom score for the UC patients.

Group	V1	V2	V3	P_V1V2_	P_V1V3_	P_between groups_
**AndoSan**^**™**^	5.88 (4.92–6.83)	4.71 (3.90–5.52)	4.50 (3.70–5.30)	**0.002**	**0.001**	**0.023**
M (n = 13)	6.15 (4.70–7.61)	5.08 (3.78–6.37)	5.08 (3.93–6.22)	**0.037**	**0.024**	
F (n = 11)	5.55 (4.09–7.00)	4.27 (3.19–5.36)	3.82 (2.66–4.97)	**0.031**	**0.017**	
**Placebo**	5.81 (4.81–6.80)	5.58 (4.42–6.73)	5.27 (4.28–6.26)	0.471	0.114	
M (n = 12)	6.33 (4.82–7.85)	6.42 (4.76–8.07)	5.83 (4.48–7.18)	0.878	0.309	
F (n = 14)	5.36 (3.90–6.82)	4.86 (3.15–6.56)	4.79 (3.25–6.26)	0.205	0.252	

V1; visit 1 (day 0), V2; visit 2 (day 14), V3; visit 3 (day 21). M; male, F; female.

Values are given as means and 95% confidence intervals. Paired sampled t-test for the p-values.

P between groups is measured with mixed models corrected for baseline values.

Regardless of disease activity at inclusion the patients had a reduction of symptom score (data not shown). Within the AndoSan^™^ group from visit 1 to 3, there was a significant reduction regarding stool frequency (p = 0.005), consistency (p = 0.02), and the doctor´s evaluation of disease activity (p = 0.02). For blood in stool (p = 0.08) and abdominal pain (p = 0.07) there was a trend favoring improvement. In the placebo group there were no significant improvements in these parameters, but a trend in favor of reduction of stool frequency (p = 0.07). The doctor’s evaluation of disease activity could be a source of bias in the CAI score because of lack of blinding. Therefore we also examined the symptoms without this item, and the p values for difference of scores were the same (data not shown). The patients did not report any harms or unintended effects of AndoSan^™^ in this study.

### 3.4. Fatigue Score

Firstly, age-adjusted normative fatigue scores in the Norwegian population were compared with such scores in the UC patients at inclusion in this study (Table 3). There were for both genders a significant increase of physical, mental and total fatigue scores in the UC patients compared with the general population. This effect was more pronounced for physical fatigue than for mental fatigue.

**Table 3:** Mean fatigue scale scores. Normative data in the Norwegian population compared to patients with UC on inclusion.

	Normative data		Ulcerative colitis		P _Normative data vs UC_	
	M (n = 1112)	F (n = 1175)	M (n = 25)	F (n = 25)	M	F
**Total**	11.9 (3.9)	12.6 (4.0)	16.32 (4.7)	16.56 (5.7)	<0.0001	<0.0001
**Physical**	7.6 (3.0)	8.2 (3.2)	11.08 (3.9)	11.28 (4.2)	<0.0001	<0.0001
**Mental**	4.3 (1.4)	4.4 (1.4)	5.24 (1.4)	5.28 (1.8)	0.0009	0.0021

Normative data from the general Norwegian population.

Values are given as mean and standard deviation (SD).

Paired sample t-test for the p-values.

The scores for genders on inclusion were quite similar within and between the groups. In the AndoSan^™^ group (Table 4) for both genders the UC patients reported a significant decline in mental and total fatigue, that was more pronounced at visit 2 (day 14) vs. visit 3 (day 21).

**Table 4:** Fatigue scores for the patients with (n = 24 AndoSan^™^ and n = 26 placebo) UC.

	AndoSan^™^ group					Placebo group					
	V1	V2	V3	P_V1V2_	P_V1V3_	V1	V2	V3	P_V1V2_	P_V1V3_	P_between groups_
**TF**	16.6 (5.6)	14.1 (4.5)	15.1 (4.1)	**0.001**	**0.023**	16.3 (4.8)	16.3 (4.9)	16.9 (5.2)	1.000	0.507	**0.018**
M	16.38	14.46	15.31	**0.002**	0.141	16.25	15.00	15.83	0.325	0.764	
F	16.91	13.73	14.91	**0.039**	0.098	16.29	17.36	17.79	0.297	0.248	
**PhF**	11.1 (4.3)	9.4 (3.8)	10.4 (3.2)	**0.007**	0.128	11.2 (3.9)	11.2 (3.4)	11.7 (3.9)	0.949	0.564	**0.037**
M	10.85	9.77	10.38	**0.042**	0.436	11.33	10.42	11.00	0.366	0.765	
F	11.45	9.00	10.45	0.055	0.199	11.14	11.86	12.21	0.325	0.289	
**MF**	5.50 (1.8)	4.71 (1.3)	4.71 (1.1)	**0.002**	**0.010**	5.0 (1.3)	5.1 (1.9)	5.2 (1.6)	0.898	0.446	**0.022**
M	5.54	4.69	4.92	**0.035**	0.071	4.92	4.58	4.83	0.339	0.809	
F	5.45	4.73	4.45	**0.024**	0.076	5.14	5.50	5.57	0.455	0.254	

V1; visit 1 (day 0), V2; visit 2 (day 14), V3; visit 3 (day 21).

TF; total fatigue, PhF; physical fatigue, MF; mental fatigue.

Values are given as mean and standard deviation (SD). Paired sampled t-test for p-values.

P between groups is measured with mixed models corrected for baseline values.

When broken down into gender the reduction in mental and total fatigue was not significant at visit 3. There was, however, a significant decline in physical fatigue at visit 2 (p = 0.007), but not at visit 3 (p = 0.128). The improvement in physical fatigue at visit 2 was significant for men (0.042) but not for women (0.055). In the placebo group the fatigue scores were unaltered throughout the experimental period as a whole and by division into gender.

However, when comparing the AndoSan^™^ and placebo groups using mixed models corrected for baseline values, there were significant improvements in the AndoSan^™^ group for the three scores of total (p = 0.018), mental (p = 0.022) and physical (p = 0.037) fatigue (Table 4).

### 3.5. Quality of Life

HRQoL score (SF-36) for UC patients were compared with age-adjusted normative data for the Norwegian population (Table 5). We found significantly much lower scores for HRQoL in the UC patients for both genders regarding 7 out of 8 dimensions. Only for physical functioning (PF) there were no differences in both genders relative to the general population. The reduction of quality life scores in the UC patients were for both genders most pronounced for the dimensions; general health (GH), vitality (VT) and social functioning (SF) (p values < 0.0001) as compared with the general population.

**Table 5:** Mean SF-36 scale scores. Age-adjusted Normative Data from the Norwegian Population compared with patients with UC on inclusion.

	Normative data		Ulcerative colitis		P _Normative data vs UC_	
	M (n = 977–1017)	F (n = 1013–67)	M (n = 25)	F (n = 25)	M	F
**PF**	91.4 (16.2)	87.7 (17.5)	85.8 (17.5)	85.6 (14.9)	0.09	0.55
**RP**	83.3 (32.0)	79.2 (35.0)	59.0 (41.4)	49.0 (40.5)	0.0002	<0.0001
**BP**	78.1 (24.6)	74.5 (26.0)	59.3 (26.0)	57.2 (23.0)	0.0002	0.0011
**GH**	78.3 (21.0)	77.5 (22.1)	51.6 (21.3)	54.4 (24.9)	<0.0001	<0.0001
**VT**	63.4 (18.2)	57.6 (21.0)	39.0 (18.6)	38.1 (19.7)	<0.0001	<0.0001
**SF**	88.2 (20.5)	84.8 (22.8)	67.5 (27.2)	66.0 (20.6)	<0.0001	<0.0001
**RE**	85.9 (28.5)	80.9 (33.1)	66.7 (41.9)	62.7 (43.4)	0.0010	0.0070
**MH**	79.7 (15.8)	77.6 (16.9)	63.5 (17.2)	68.6 (13.6)	<0.0001	0.0082

Age-adjusted normative data from the general Norwegian population, age 19–69.

Values are given as mean and standard deviation (SD). Paired sampled t-test for the p-values.

SF-36; Short form 36, PF; physical functioning, RP; role limitations, physical, BP; bodily pain, GH; general heath perception, VT; Vitality, SF; social functioning, RE; role limitations, emotional, MH; mental health.

In the AndoSan^™^ group as a whole the results were significantly improved (Table 6) for bodily pain (BP), VT, SF and mental health (MH), of which BP and MH also scored significantly but less pronounced at visit 2. Although not significant, there was an increase of scores for the remaining four dimensions GH, PF, role limitation, physical (RP) and role limitation, emotional (RE). In the placebo group, except from an improvement of BP (p = 0.036) at visit 2, there were no significant alterations in the 8 quality of life dimensions throughout the study. When comparing the two groups, using mixed models corrected for baseline values, we found significant improvement of SF-36 scores for PF, RP, BP and SF in the AndoSan^™^ group.

**Table 6:** Mean SF-36 scale scores (n = 24 AndoSan^™^ and n = 26 placebo) UC.

	AndoSan^™^ group					Placebo group					
	V1	V2	V3	P_V1V2_	P_V1V3_	V1	V2	V3	P_V1V2_	P_V1V3_	P _AvsP_
**PF**	85.0 (16.9)	87.6 (13.7)	88.8 (13.4)	0.096	0.101	86.4 (15.5)	85.8 (14.6)	84.6 (15.5)	0.701	0.265	**0.039**
M	86.9	89.9	90.4	0.148	0.145	84.6	85.0	84.2	0.809	0.045	
F	82.7	84.9	86.8	0.399	0.347	87.9	86.4	85.0	0.569	0.220	
**RP**	55.2 (43.0)	59.4 (39.6)	64.6 (40.3)	0.426	0.059	52.9 (39.6)	47.1 (43.2)	47.1 (43.8)	0.265	0.265	**0.028**
M	61.5	71.2	69.2	0.096	0.219	56.3	60.4	58.3	0.551	0.586	
F	47.7	45.5	59.1	0.810	0.176	50.0	35.7	37.5	0.055	0.169	
**BP**	57.1 (26.1)	67.3 (24.0)	73.6 (21.5)	**0.015**	**0.000**	59.3 (23.0)	64.7 (25.0)	58.3 (62.0)	**0.036**	0.760	**0.013**
M	59.3	70.8	77.8	0.114	**0.015**	59.3	68.5	61.9	**0.009**	0.655	
F	54.5	63.2	68.7	**0.022**	**0.010**	59.4	61.5	55.3	0.567	0.281	
**GH**	53.0 (23.2)	56.5 (24.0)	54.8 (22.2)	0.174	0.536	52.9 (23.1)	50.7 (23.4)	48.7 (21.1)	0.299	0.093	0.062
M	49.5	55.8	53.5	0.137	0.374	53.8	55.7	53.6	0.343	0.936	
F	57.3	57.4	56.4	0.972	0.789	52.1	46.5	44.6	0.107	**0.046**	
**VT**	38.8 (19.9)	44.6 (18.9)	46.9 (17.9)	0.076	**0.018**	38.4 (18.5)	40.6 (19.4)	38.3 (17.0)	0.403	0.968	0.088
M	39.2	46.9	48.1	0.139	**0.046**	38.8	47.1	42.9	**0.014**	0.248	
F	38.2	41.8	45.5	0.371	0.205	38.1	35.0	34.3	0.406	0.466	
**SF**	65.1 (26.6)	75.5 (22.9)	75.0 (19.8)	**0.013**	**0.015**	66.3 (21.6)	65.9 (21.1)	67.8 (20.7)	0.597	0.885	**0.014**
M	67.3	81.7	75.0	**0.050**	0.180	67.7	75.0	68.8	0.306	0.870	
F	62.5	68.2	75.0	0.096	**0.041**	68.8	58.0	67.0	0.061	0.583	
**RE**	68.1 (39.9)	69.4 (40.4)	75.0 (39.6)	0.846	0.423	61.5 (44.9)	52.6 (44.4)	55.1 (43.1)	0.215	0.477	0.095
M	64.1	64.1	71.8	1.000	0.461	69.4	72.2	77.8	0.809	0.555	
F	72.7	75.8	78.8	0.588	0.690	54.8	35.7	35.7	0.040	0.104	
**MH**	65.3 (15.7)	69.5 (14.4)	71.0 (13.7)	**0.032**	**0.005**	66.8 (15.7)	69.2 (17.1)	66.0 (17.1)	0.285	0.671	0.123
M	63.1	67.4	68.0	0.141	0.075	64.0	70.7	66.3	**0.041**	0.385	
F	68.0	72.0	74.5	0.137	**0.036**	69.1	68.0	65.7	0.723	0.165	

Paired sampled t-test for the p-values.

P between groups is measured with mixed models corrected for baseline values.

Except for unaltered score in GH for females in the AndoSan^™^ group, there were improvements of scores in all dimensions for both genders. In the placebo group, however, the corresponding general pattern was a gender-like unchanged score during the three weeks study period (Table 6).

### 3.6. Calprotectin in Feces

The patients delivered fecal tests at visits 1, 2 and 3 (Table 7). In the AndoSan^™^ group the median (range) values for fecal calprotectin (mg/kg) were 439 (10–6000), 366 (10–6000) and 489 (18–6000). In the placebo group the values were 328 (16–5361), 521 (18–6000) and 563 (10–6000). There were no significant differences in levels of calprotectin within or between the groups. However, for men there was rather a significant increase of calprotectin in the placebo group from visit 1 to visit 3 (p = 0.019).

**Table 7:** Fecal test in the 50 UC patients.

Group	V1	V2	V3	P_V1V2_	P_V1V3_	P_between groups_
**AndoSan**^**™**^ (n = 24)	439 (10–6000)	366 (10–6000)	489 (18–6000)	0.673	0.808	0.705
M	358	382	527	0.727	0.600	
F	517	317	366	0.314	0.959	
**Placebo** (n = 26)	328 (16–5361)	521 (18–6000)	563 (10–6000)	0.298	0.551	
M	234	570	1702	0.060	**0.019**	
F	679	480	445	0.875	0.245	

Values are given as median (range). Non-parametric Wilcoxon test for the p-values.

P between groups is measured with Mann-Whitney U test.

### 3.7. Effect on General Blood Parameters

The following blood samples were analyzed at visit 1 and 3: CRP, leukocytes, eosinophils, basophils, neutrophils, lymphocytes, monocytes, hemoglobin, haematocrite, mean cellular volume, mean cellular haemoglobin, immature reticulocytes, reticulocytes, thrombocytes, urea, creatinine, and GFR (glomerular filtration rate), bilirubin, aspartate aminotransferase, alanine aminotransferase, lactate dehydrogenase, γ-glutamine transferase, alkaline phosphatase and pancreatic amylase. Significant changes in the blood samples were neither found in the AndoSan^™^—nor the placebo group.

The median and range haemoglobin (g/l), leukocyte counts (10^9^/l) and CRP levels for visit 1 and 3 the AndoSan^™^ group were 13.55 (11.5–17.2) versus 13.55 (11.0–17.3), 6.20 (3.1–9.2) versus 5.95 (3.3–10.5) and 2.00 (0.6–30.4) versus 1.80 (0.6–16.9), respectively. Corresponding values in the placebo group were 13.7 (5.7–16.1) versus 13.5 (6.0–15.8) for haemoglobin, 5.85 (4,4–10.3) versus 6.55 (3.1–14.6) for leukocytes and 1.60 (0.6–63) versus 1.95 (0.6–58.0) for CRP. Accordingly, there were no statistical changes of these parameters neither within nor between the groups.

## 4. Discussion

The present study demonstrates for the first time, in a randomized patient-blinded placebo controlled study, that the immunomodulatory Agaricus *blazei* Murill based mushroom extract AndoSan^™^ [14] improved clinical symptoms as well as fatigue and quality of life in patients with UC during a 3 weeks’ study period. Moreover, there was no effect in the placebo group, whatsoever, and when comparing the two groups the difference was significant in favor of AndoSan^™^. The moderate, but significant reduction (about 20%) in symptom score occurred already after 2 weeks and persisted after 3 weeks.

Compared with the normal population the UC patients had considerably more fatigue (women 32%, men 37%). However, the general effect on fatigue subsided after 3 weeks but still was significant for mental fatigue. This may be due to a reduced effect of AndoSan^™^ over time, but external factors influencing the patients’ physical status like intercurrent subclinical disease must also be considered.

Regarding limitations of this study we need to adress some issues. The study was not blinded for the authors leaving possible biases of the results. This is especially true for the first author who was responsible for the inclusion and randomization of participants, the implementation of the practical aspects of and in meeting with the patients, and also in the analysis of the results. This is a relatively small study, allthough with significant results, with its limitations. The study was conducted with participants from the Oslo (Norway) area, and the results may not be transferable to other geographical areas with other patient characteristics. Reduced compliance in carrying out the study, with missing or incorrect oral intake of AndoSan^™^ or placebo, may be a possible source of error, even though this was not the impression in conversation with patients after the study period of three weeks. The modified CAI is not used much in previous studies, making it somewhat difficult to compare our results with previous studies done on this parameter.

For HRQoL in the AndoSan^™^ group, which is a broad coverage of the patient’s well-being, there was a persistent an even improved effect on bodily pain (29% reduction), vitality (21%), social functioning (15%) and mental health (9%). Although no within-group significance for physical functioning and role limitation, there was a significant effect in favor of the AndoSan^™^ compared with the placebo group.

In line with a previous study [34] in which 10 UC patients received the same amount of AndoSan^™^ for 12 days, there were no alterations in general blood parameters including CRP. However, contrary to this study there was a significant reduction of fecal calprotectin in those patients during the study period. However, in the placebo group for men in the present study there was, on the other hand, a significant increase in level of fecal calprotectin (p = 0,019), implying a prospective stabilizing effect on calprotectin levels in the AndoSan^™^ group that was not seen for the controls. One reason for lack of reduction of fecal calprotectin in this study could be the large variability of baseline calprotectin levels (range 10–6000) that was not seen in the previous small pilot study (128–1683).

The AndoSan^™^ and placebo groups had similar baseline values with respect to symptom score, fatigue and quality of life. The groups were also comparable with regard to disease duration, extent of disease, disease pattern, arthralgia and relevant comorbidity, and thereby well-fitted for comparison. Hence, putatively increased symptoms tolerance in patients with extensive or prolonged disease experience, should not affect the SF-36 results.

The patients did not report any obvious clinical side-effects from consumption of the mushroom extract, which is similar to clinical studies in patients with chronic hepatitis B [40] or hepatitis C infection [24] where liver function was either normalized or unaltered. However, although other causative factors such as cancer chemotherapy and hepatitis virus could not be ruled out, consumption of Agaricus *blazei* extract for days to months may have induced severe hepatic dysfunction in three patients receiving concomitant chemotherapy for breast and ovarian cancer [41]. In a phase I study different doses (1.8 g, 3.6 g and 5.8 g) of AbM granulated powder was consumed in orally for 6 months in 78 patients with different cancers [42]. Adverse effects were observed in 12%, mainly nausea and diarrhea. Only in one case with ovarian cancer receiving six cycles of chemotherapy (Paclitaxel/Carboplatin) after surgery, generalized urticaria with papulae and moderate liver dysfunction occurred after two months’ AbM consumption, which was the definitive cause as judged by a positive lymphocyte AbM stimulation test. However, in another recent clinical trial done with AndoSan^™^ or the same placebo as used here, as supplementary treatment 60 ml/day over 7 weeks to high-dose chemotherapy and bone marrow transplantation for 40 patients with multiple myeloma, there were no side effects, neither during nor after the trial [43]. Hence, AndoSan^™^ is proven to be a safe product *per se* for very different categories of patients. However, caution is advised for possible interaction with some drugs as referred below.

Herb-drug interactions are associated with cytochrome P-450 metabolism in the liver and the trans-membrane efflux pump P-glycoprotein (P-gp) that is present in normal intestinal lumen where it may limit drug absorption, as well as in the liver, where it may increase excretion of the drug [44]. In this respect, AndoSan^™^ (called Agaricus from Japan) has previously been investigated by another independent researchers at the Department of Cancer Research and Molecular Medicine, Norwegian University of Science and Technology, for *in vitro* inhibitory potential on P-gp-mediated transport in an intestinal cell line. It was found that AndoSan^™^ inhibited P-gp in vitro in a similarly as did green tea. Because the mushroom may interact with some drugs beeing P-gp substrates (i.e. vinblastine, loperamide, digitoxin, cyclosporine, verapamil) concomitant AbM should not be given to patients using these drugs. When tested in vitro for inhibition potential on cytochrome P-450 (CYP3A4 isoform) the AndoSan^™^ extract was found to inhibit it but far less than green tea [45]. The researchers [45] concluded that “clinical relevant systemic or intestinal interactions with CYP3A4 were considered unlikely”. In our clinical study, none of the UC patients were concomitantly treated with the above mentioned anticancer-, heart-, immunodepressive- or diarrhea drugs.

The notion of a potential anti-inflammatory effect after intake of AndoSan^™^ was a result of the surprising finding of reduced serum pro-inflammatory cytokines (IL-1ß, IL-6, IL-8) and chemokines (MCP-1, G-CSF, GM-CSF) in a pilot safety study with AndoSan^™^ in healthy individuals without a placebo control [25]. Recently, a steroid 4-hydroxy-17-methylincisterol (4-HM) [12] isolated from AbM dose-dependently suppressed the synthesis in PHA-stimulated peripheral blood mononuclear cells of cytokines IL-2, IL-4, IFNγ and TNFα by decreasing both NF-AT (nuclear factor of activated T-cells), which belongs to a family of transcription factors required for activation and proliferation of T lymphocytes including production of the first three aforementioned cytokines, and NF-κB—the latter being the “mother” of all inflammation. Substances isolated from AbM had several anti-inflammatory effects in rats, related to IL-1β, TNFα and IL-8 modulations [11,46]. In a study on healthy volunteers ingesting AndoSan^™^ [47] there was a reduction *in vivo* of ROS mainly reflecting superoxide ions, and again pointing to an anti-inflammatory effect. However, this result was not demonstrated in the UC patients. The reason for reduced superoxide anions may be related to reduction of IL-1ß because inhibitors of ROS reduce synthesis of this cytokine in macrophages [48].

There also was an anti-allergic effect in mice sensitized to ovalbumin (OVA), regarding reduction of specific anti-OVA IgE antibodies, both when AndoSan^™^ was given before or after the OVA immunization [49]. Additionally, in this allergy model there was an increase in Th1 relative to Th2 cytokines in spleen cell cultures ex vivo obtained from the animals treated with AndoSan^™^ [49]. Moreover, the inhibitory effect of an isolated carbohydrate fraction of AndoSan^™^ [22] on the tissue degrading enzyme legumain (aspariginyl endopeptidase), which probably activates proMMP and processing of cathepsins may also contribute to less pro-inflammatory activity in the UC patients.

It is commonly believed that carbohydrates larger than monosaccharides are not absorbed from the human gut. However, in murine models [13, 50], uptake of β-1,3 glucans across the gut wall, probably by microfold cells (M cells) but also by dendritic cells (DC) [51], has been demonstrated. The β-glucan may further be transported by DC to lymphocytes in GALT, but also circulated in blood in rodents [52, 53]. Presumably, a similar mechanism is operating in humans for intestinal absorption of small immunomodulatory bioactive β-glucan fragments into the lymphoid system and blood. As mentioned, other yet unidentified small immunomodulatory substances in the mixed mushroom extract are probably also playing a role in this context. This assumption is supported by the fact that when molecules <12.5 kDa, and thus smaller than β-glucans, were dialyzed away from the AndoSan^™^ extract prior to performing the experiments in the mouse allergy model, the anti-allergic effect of the extract was diminished to no longer statistically significant levels [49]. Hence, since the anti-allergic effect of AbM extract seems to be owing to low-molecular-weight substances in the mouse allergy model, other smaller and simpler substances than ß-glucans in AbM could very well be as important for the mushroom’s biological effect in other settings such as UC. In addition, AbM also contains small molecular anti-oxidant and anti-inflammatory substances that may be absorbed actively or by diffusion through the enterocytes in the gut. A key to understanding the function of a changed or down-regulated cytokine response locally in the gut-wall, is probably the signals mediated by DC [54] after processing and presenting to CD4 T helper cells of native antigens from the mushrooms or novel bacteria-derived antigens resulting from mushroom-microbiota interactions. As seen from animal models [49, 55], the AbM extracts seem to drive the Th1/Th2 balance towards an increased Th1 response, which also inhibits Treg cells and Th17 cells [14].

In conclusion, the results support that AndoSan^™^ may be beneficial as a supplement to conventional medication in UC patients with mild to moderate disease activity because of improvements in symptoms, fatigue and quality of life. There is reason to assume that AndoSan^™^ may stabilize these patients with subsequently less need for increased medical treatment, which may be associated with potentially troublesome and harmful side effects.

## Supporting Information

S1 Fig Flow chart UC kopi. (TIF)

S1 CONSORT Checklist CONSORT 2010 Checklist. (DOC)

S1 Protocol Study Protocol Norwegian/English version. (DOCX)

S2 Protocol Study Protocol English version. (DOCX)
