# Supplementary material for: Effect of a Medicinal Agaricus blazei Murill-Based Mushroom Extract, AndoSan™, on Symptoms, Fatigue and Quality of Life in Patients with Ulcerative Colitis in a Randomized Single-Blinded Placebo Controlled Study
Source: PLoS One. 2016 Mar 2;11(3):e0150191. doi: 10.1371/journal.pone.0150191 (PMC4774976; doi:10.1371/journal.pone.0150191)
Supplement: S1 Protocol — (DOCX) [file pone.0150191.s003.docx]

**PREKVALIFISERING, SKJEMA (MAL) FOR PROSJEKTSØKNAD**

Søker: Egil Johnson Kinikk/Avdeling: Kreft-, kirurgi- og transplantaskjonsklinikken/ Gastro- og barnekirurgisk avd., Oslo universitetssykehus, Ullevål HF

E-mail: [egil.johnson@medisin.uio.no](mailto:egil.johnson@medisin.uio.no) Mobil: 47416334

**_____________________________________________________________________________**

**Prosjekttittel:** Anti-inflammatory effect of a mushroom extract (AndoSan^TM^) in patients with inflammatory bowel disease. A prospective blinded randomised study.

**Studiens hoved- og delmål:**

**Hovedmål:** Examine whether daily oral ingestion of a immunomodulatory mushroom extract (AndoSan^TM^) in patients with ulcerative colitis (UC) and Crohn`s disease (CD), experience clinical, biochemical and genetical improvement in their disease.

**Delmål:**

1. Compare prior to (day 0), during (day 14) and after (day 21) ingestion of AndoSan^TM^  (daily 20 ml x 3) whether benefitial effects has occurred concerning:
2. general blood samples (leucocytes,CRP, liver and renal function tests)
3. level of blood cytokines and analytes (IL-1α/-1ra/-2/-4/-5/-6/-7/-8/-9/-10/-12(p70)/-13/-15/-17,G-CSF,GM-CSF,MCP-1,MIP-1ß/-1α,IFNγ,TNFα and basic FGF, eotaxin, PDGF-BB, RANTES, VEGF)
4. level of inflammatory marke calprotectin in feces and blood in patients with UC and CD
5. genetic expression in blood leukocytes (microarray)
6. clinical symptom score; simple clinical colits activity index (SCCAI) for UC and simplified Harvey-Bradshaw index for CD (SCDAI)
7. life quality (SF-36 version 2) and fatigue scores
8. rectosigmoidoscopy before (day 0) and after ingestion of AndoSan^TM^ (day 21) in patients with UC

**3. Prosjektbeskrivelse**

**3.1 Bakgrunn og vitenskapelig rasjonale for studien:**

The *Agaricus blazei* Murill mushroom (AbM) (jap.: Himematsutake) of the *Basidiomycetes* family grows wildly in the coastal Piedade area outside of São Paulo, Brazil. People in this area have traditionally used AbM as a health food ingredient. The frequency of serious diseases like atherosclerosis, hepatitis, hyperlipidemia, diabetes and cancer were lower in Piedade than in neighbouring regions, supposedly due to the AbM intake. In 1966 the mushroom was taken to Japan and introduced to the health food market, and later AbM was also subjected to an increasing research effort. Other interesting medicinal *Basidiomycetes* mushrooms that have been studied are *Grifola frondosa* (Gf) (Maitake) and *Hericeum erinaceum* (He) Yamabushitake). AbM, like Gf, is rich in immunostimulatory mixture of β(1-3)-, β(1-4) and β(1-6)-D-glucans with antitumor activity, probably secondary to modulation of NK-cells and monocytes / macrophages of native immunity. *In vitro* AbM stimulates mononuclear phagocytes to secrete nitric oxide and pro-inflammatory cytokines like IL-1β, IL-6, and TNFα, and chemokine IL-8. The AbM based extract Andosan^TM^ which we have used in several studies, is composed of 82% AbM, 15% He and 3% Gf. Recently, the stimulatory effect of Andosan^TM^ on cytokine production (TNFα, IL-1ß, IL-6, IL-8, G-CSF and MIP-1ß) in monocyte-derived dendritic cells has also been demonstrated [1]. The effects are probably mediated by binding of sugars in AbM to Toll-like receptor-2 (TLR-2), but also to dectin-1 and the lectin-binding site of CD11b/18 and possibly CD11c/18. Gene microarray expression analysis of promonocytic THP-1 tumor cells supported these results because stimulation with AbM strongly upregulated genes for IL-1β and IL-8, moderately for TLR-2 and co-operative molecule MyD88, but not for TLR-4. On the other hand, daily consumption of 60 ml Andosan^TM^ for seven days in patients with chronic hepatitis C, had no effect *in vivo* on the expression of these genes in blood cells.

AbM is reported to have antitumor properties in mouse models against fibrosarcoma, myeloma, ovarian-, lung- and prostate cancer, and in human studies against gynecological cancer (increased NK-cell activity and quality of life) and leukemia. Moreover, AndoSan^TM^ given orally is shown to increase survival from bacterial sepsis in mice inoculated i.p. a day after with pneumococci or fecal bacteria. The mushroom extract also protected against IgE-mediated allergy in a mouse model when given p.o. [all above reviewed in references 2 and 3].

Recently, we reported that Andosan^TM^ stimulation of whole blood *ex vivo* [4] stimulated the release of all the 17 different cytokines, chemokines and leukocyte growth factors tested. The cytokines were pro-inflammatory (IL-1β, IL-6, TNFα), anti-inflammatory (IL-10), pleiotropic (IL-7, IL-17) and of the Th1- (IFNγ, IL-2, IL-12) and Th2-types (IL-4, IL-5, IL-13), chemokines IL-8, MIP-1β, MCP-1 and leukocyte growth factors G-CSF and GM-CSF. On the other hand, when blood was collected from volunteers prior to and 12 days after their daily intake (60 ml) of Andosan^TM^, there was *in vivo* either a significant reduction in cytokine levels for IL-1β, TNFα, IL-6, IL-2 and IL-17 or unaltered levels of the remaining twelve factors. This pointed to a stabilising and anti-inflammatory effect of AbM *in vivo* when given via the oral route.

Inflammatory bowel disease (IBD) like UC and CD are bothersome conditions of unknown etiology with a prevalence of about 20 and 10 per 100.000 people, respectively. The treatment is based on a combination of different drugs (prednisolone, 5-ASA, sulpha-salazine, azathioprin, infliximab, adalimumab) and surgery. Crucial symptoms are diarrhea, anemia, joint pain, weight loss, fistulization and abscess formation. There is no definitive healing treatment and medication exerts bothersome side effects in these patients, including deteriorated tissue repair, tissue atrophy, peptic ulcer, superinfection and anaphylactoid reactions. Patients with UC and CD have in the colon mucosa an unselective increase in chemokine expression including that of MIP1-ß, MCP-1 and IL-8 as well as cytokines IL-1ß, IL-6 and TNFα. Cytokine levels in serum, however, are less extensively studied, but increased levels of IL-6 and TNFα have been detected in patients with UC and CD. Recently, increased serum levels of the chemokine MIP-1ß were found in patients with UC. Based on the anti-inflammatory and stabilising effect of the AbM-based mushroom extract AndoSan^TM^ on cytokine release in blood *in vivo* and *ex vivo* in healthy volunteers after 12 days consumption [4], we investigated whether the same effect was valid in patients with UC and CD [5]. In addition, calprotectin an abundant cytosolic protein in neutrophils and a surrogate marker for degree of intestinal inflammation, was measured in blood and feces of these patients. After 12 days ingestion of AndoSan^TM^ baseline plasma cytokine levels in UC was reduced for MCP-1 (40%) and in LPS-stimulated blood for MIP-1ß (78%), IL-6 (44%), IL-1ß (41%), IL-8 (30%), G-CSF (29%), MCP-1 (18%), and GM-CSF (17%). Corresponding reductions in CD were for IL-2 (100%), IL-17 (55%) and IL-8 (29%), and for IL-1ß (35%), MIP-1ß (30%), MCP-1 (22%), IL-8 (18%), IL-17 (17%) and G-CSF (14%), respectively. Fecal calprotectin was reduced in the UC group. The IBD patients also reported spontaneously reduction in bowel movements. In conclusion, ingestion of an AbM-based medicinal mushroom by IBD patients resulted in interesting anti-inflammatory effects as already described (Ph. D. thesis 2011 by Dag. T. Førland). Accordingly, use of AndoSan^TM^ in patients with IBD may function as a support therapy to improve the patients’ symptoms and reduce their need for conventional medical therapy. The next mandatory step is to perform a two armed double-blind randomised study comparing use of AndoSan^TM^ versus placebo for 4 weeks for parameters stated under partial aims (1. a-g).

**3.2 Materialer og metoder (event. inkl. statistisk styrkeberegning):**

ELISA based assays for measurement of 27-plex cytokines and calprotectin. Analysis of general blood samples. Use of validated symptom scores for UC and CD and SF-36 life quality form (version 2). Blinded randomisation of patients into experimental group given Andosan^TM^  and placebo group given placebo. Analysis of parameters prior to (day 0), during (day 12) and after (day 24) consumption of additives in doses 20 ml thrice daily. Prospective difference of 20% between the experimental group and placebo group and assumed standard deviation of 20% for the different parameters with a significance level of 5% (α<0.05) and a power of 90% (ß =0.10), demands about 25 persons per randomised arm (calculated in cooperation with Biostatistics and Epidemiology Unit, Oslo University Hospital). Non-parametric Mann-Whitney and Wilcoxon paired sample tests are used for comparison between the groups and within the groups, respectively, before and after intervention (biochemical tests). Corresponding clinical variables (symptom score and life quality score) are evaluated by parametric Student’s t-test and paired Student’s t-test, respectively.

1. **Status for prosjektet ved søknadsfrist og fremdriftsplan**

2011: The project was approved by the regional ethical committee.

November 2011 to December 2012: Recruitment of patients for experimental work.

January 2013-2015: Manuscript formation and delivery of thesis (Ph. D.).

1. **Samarbeidende forskningsgrupper i inn- og utland (gjerne i tabellform):**

| Dr. Stig Palm Therkelsen, Gastrokir. avd., OUS, Ullevål, Oslo |
| --- |
| Overlege dr. med. Geir Hetland, Avd. for Celleterapi, OUS, Radiumhospitalet, Oslo |
| Avd. overlege dr. med. Idar Lygren, Gastromed. avd., OUS, Ullevål, Oslo |
| Forskningsleder dr. med. Torstein Lyberg, OUS, Ullevål, Oslo |
| Seksjonsoverlege dr. med Jørgen Jahnsen, Gastromed. Avd., OUS, Ullevål, Oslo |

1. **Litteratur (mest sentrale referanser):**
2. Førland DT, Johnson E, Tryggestad AMA, Lyberg T, Hetland G. An extract based on the medicinal mushroom *Agaricus blazei* Murill stimulates monocyte-derived dendritic cells to cytokine and chemokine production *in vitro*. *Cytokine* 2010;49:245-50.
3. Hetland G, Johnson E, Lyberg T, Bernardshaw S, Tryggestad AMA, Grinde B. Effects of the medicinal mushroom *Agaricus blazei* Murill on immunity, infection and cancer. Review. *Scand J Immunol* 2008; 68: 363-70.
4. Firenzuoli F, Gori L, Lombardo G. The medical mushroom *Agaricus blazei* Murill:

review of literature and pharmaco-toxicological problems. *Evid Based Complement*

*Alternat Med* 2008;5:3-15.

1. Johnson E, Førland DT, Sætre L, Bernardshaw SV, Lyberg T, Hetland G. Effect of an extract based on the medicinal mushroom *Agaricus blazei* Murill on release of cytokines, chemokines and leukocyte growth factors in human blood *ex vivo* and *in vivo*. *Scand J Immunol*  2009;69:242-50.
2. Førland DT, Johnson E, Sætre L, Lyberg T, Lygren I, Hetland G. Effect of an extract based on the medicinal mushroom *Agaricus blazei* Murill on expression of cytokines and calprotectin in patients with ulcerative colitis and Crohn’s disease. Scand J Immunol 2011;73:66-75.

# 1. Curriculum vitae for Egil Johnson

**CV**

I am born 1955, exchange student in 1972-73 in USA, finished secondary school 1975, cand. med. Universitetet i Tromsø 1982. Special consultant in general surgery 1992, gastro-enterological surgery 1994. Consultant in gastroenterological surgery at Ulleval hospital since 1995 and also professor since 1999. Doctor of medicine 1983. Thesis “Glycan stimulation of macrophages in vitro”, University of Tromsø.

**Scientific activity**

Performed basal and clinical research since 1982. Cellular work dealing with complement biosynthesis and endocytosis, CD antigens, production of cytokines and reactive oxygen species. Translational work dealing with anti-inflammatory effect of a mushroom extract on healthy individuals and patients with inflammatory bowel disease (CD and UC). Clinical studies including anal incontinence and fissure, rectal prolapse,CD and UC, oesophageal cancer and perforation, neutropenic enterocolitis, laparoscopic appendectomy and cholecystectomy. Reviews on complement biosynthesis, effects of a mushroom extract, pathogenetic aspects in sarcoidosis etc.

**Supervision**

Main supervisor for five candidates for doctor of medicine/Ph.D.. In 1990 (Helge Bjørnstad Pettersen, University of Trondheim), 1997 (Viktor Berge, University of Oslo), 2003 (Harald Langeggen, University of Oslo) og 2007 (Soosaipillai Bernardshaw, University of Oslo) and Dag Tidemann Førland (December 2011). Assistant supervisor for one candidate for doctor of medicine in 1987 (Geir Hetland, University of Tromsø).

**Supervision (students)**

Supervised four medical students for compulsory turnpaper.

**Presentations**

Presentations of medical topics at national, nordic and international meetings. Moderator at national and nordic meetings.

## Committee work

Since 1994 participated in 24 committees concerning evaluation of candidates for the degree doctor of medicine/Ph.D., qualification for professorship and different national committees related mainly to the field of surgery.

**Course responsible**

Leader of the PP-kurset since 2010, and participated since 2008. Organised Nordic Postgraduate Course in Colorectal Surgery in 2010, participated since 2002.

# Publications last 5 years

# Original publications (english journal)

A 52. Johnson E., Enden T., Noreng H.J., Holck-Steen A., Gjerlaug B.E., Morken T.,

Johannessen H.-O., Drolsum A. Survival and complications after insertion of self-

expandable metal stents for malignant oesophageal stenosis. Scand J Gastroenterol

2006; 41 (3): 252-56.

A 53. Skjelbred CF, Saebo M, Wallin H, Nexo BA, Hagen PC, Aase S, Johnson E, Hansteen

IL, Vogel U, Kure EH. Polymorphisms of the XRCC1, XRCC3 and XPD genes and

risk of colorectal adenoma and carcinoma, in a Norwegian cohort: a case control study.

adenomas and colorectal cancer. BMC cancer 2006; 6:67.

A 54. Bernardshaw S, Hetland G, Grinde B, Johnson E. An extract of the mushroom

*Agaricus Blazei* Murill protects against lethal septicaemia in a mouse model for fecal

peritonitis. Shock 25: 420-25, 2006.

A 55. Ellertsen L, Hetland G, Johnson E. Effect of a medicinal extract from *Agaricus blazei*

Murill on gene expression in human monocytes. Int Immunopharmacol 2006;6 (2):133-

43.

A 56. Grinde B, Hetland G, Johnson E. Effects on gene expression and viral load of a

medicinal extract from Agaricus blazei in patients with chronic hepatitis C infection.

Int Immunopharmacol 2006; 6 (8): 1311-14.

A 57. Landsend E, Johnson E, Johannessen H-O, Carlsen E. Long-term outcome after

intestinal resection for Crohn’s disease. Scand J Gastroenterol 2006; 41 (10): 1204-08.

A. 58. Johnson E, Stangeland A, Johannessen H-O, Carlsen E. Resection rectopexy for

external rectal prolapse reduces constipation and anal incontinence. Scand J Surg 2007;

96 (1): 56-61.

A 59. Bernardshaw SV, Lyberg T, Hetland G, Johnson E. The effect of an extract of the

mushroom Agaricus blazei Murill on expression of adhesion molecules and production

of reactive oxygen species in monocytes and granulocytes in whole blood ex vivo.

APMIS 2007; 115: 719-25.

A 60. Sæbø M, Skjelbred CF, Brekke Li K, Bowitz Lothe IM, Hagen PC, Johnson E, Tveit

KM, Kure EH. CYP1*A2*164 A→C polymorphism, cigarette smoking, consumption of

well –done red meat and risk of developing colorectal adenomas and carcinomas.

Anticancer Research 2008;28:2289-96.

# A61. Hetland G, Johnson E, Lyberg T, Bernardshaw S, Tryggestad AMA, Grinde B. Effects

# on the medicinal mushroom *Agaricus blazei* Murill on immunity, infection and cancer.

# Scand J Immunol 2008;68:363-70.

# A 62. Johnson E, Førland DT, Sætre L, Bernardshaw SV, Lyberg T, Hetland G. Effect of an

# extract based on the medicinal mushroom *Agaricus blazei* Murill on release of

# cytokines, chemokines and leukocyte growth factors in human blood *ex vivo* and in

# vivo. Scand J Immunol 2009;69:242-50.

A 63. Førland DT, Johnson E, Tryggestad AMA, Lyberg T, Hetland G. An extract based on

the medicinal mushroom *Agaricus blazei* Murill stimulates monocyte-derived

dendritic cells to cytokine and chemokine production *in vitro*. Cytokine 2010;49:245-

50.

A 64. Gondal G, Paulsen V, Johnson E. Behandling av nøytropen enterokolitt. Tidsskr Nor

Legeforen 2010;130 (2):143-45.

A 65. Andersson T, Lunde OC, Johnson E, Moum T, Nesbakk A. Long-term functional

Outcome and quality of life after proctocolectomy for ileal puch-anal anastomosis.

Colorectal Disease DOI:10 1111/j.1463-1318 2009.o2163.x.

A 66. Førland DT, Johnson E, Sætre L, Lyberg T, Lygren I, Hetland G. Effect of an extract

based on the medicinal mushroom *Agaricus blazei* Murill on expression of cytokines

and calprotectin in patients with ulcerative colitis and Crohn’s disease. Scand J

Immunol 2011;73:66-75.

A 67. Bornholdt J, Friis S, Godiksen S, Poulsen SS, Santoni-Rugiu E, Bisgaard HC, Lothe I

MB, Ikdahl T, Tveit KM, Johnson E, Kure EH, Vogel LK. The level of claudin-7 is

reduced as an early event in colorectal carcinogenesis. BMC Cancer 2011;11:65.

A 68. Wik TA, Hjorthaug JOB, Johannessen H-O, Johnson E. Sigmoideostomy-related

parastomal hernia. Scand J Surg 2011;100:186-89.

# Review (english journal)

C3. Hetland G, Johnson E, Lyberg T, Bernardshaw S, Tryggestad AMA, Grinde B.

Effects of the medicinal mushroom *Agaricus blazei* Murill on immunity,

infection and cancer. Review. *Scand J Immunol* 2008; 68: 363-70.

C4. Hetland G, Johnson E, Lyberg T, Kvalheim G. The mushroom *Agaricus blazei* murill

elicits medicinal effects on tumor, infection, allergy and inflammation through its

modulation of innate immunity, and amelioration of Th1/Th2 imbalance and

inflammation. Pharmacological Sciences, vol. 2011, Article ID 157015, 10

pages, 2001. Doi:10.1155/2011/157015.

# Original publications (norwegian journal)

A 17. Behandling av øsofagusperforasjoner. Gondahl G, Johnson E, Johannessen H-O,

Hofstad B. Tidsskr Nor Lægeforen 2008; 128 (9): 1050-52.

A 18. Gondal G, Paulsen V, Johnson E. Behandling av nøytropen enterokolitt. Tidsskr Nor

Legeforen 2010;130 (2):143-45.

A 19. Hauge T, Johnson E, Løberg EM, Johannessen HO. Reseksjon for cancer ventriculi.

Publiseres i Kirurgen Desember, 2011.

**Medical news**

1. Hetland, G, Johnson E. Matsopp virker immunmodulerende. Tidsskr Nor Legeforen 2010; 130 (5):466.

**_____________________________________________________________________**

**Merk følgende:**

**Prosjektsøknad skal være på maksimalt 3 sider (12 pkt), enkel linjeavstand med vanlig marg. Følgende vedlegg skal være med:**

- **CV (maks 3 sider), inkludert liste over PhD-grader med hoved- eller medveilederskap**
- **Publikasjonsliste siste fem år (kun originalartikler og bokkapitler i referee-baserte tidsskrifter og bøker)**

**Søknadspapirene må scannes inn i ett dokument (pdf-fil) og mailes til** [**personal@klinmed.uio.no**](mailto:personal@klinmed.uio.no) **innen 8.2.2011**

**SØKNADER SOM IKKE UTFORMES OG INNSENDES I HENHOLD TIL RETNINGSLINJER BLIR IKKE BEHANDLET**
